# Assessing multidisciplinary follow-up pattern efficiency and cost in follow-up care for patients in cervical spondylosis surgery: a non-randomized controlled study

**DOI:** 10.3389/fmed.2024.1354483

**Published:** 2024-04-03

**Authors:** Zhongmin Fu, Yan Xie, Peifang Li, Menghui Gao, Jiali Chen, Ning Ning

**Affiliations:** Department of Orthopedics, West China Hospital, Sichuan University, Chengdu, China

**Keywords:** multidisciplinary team, cervical spondylosis surgery, contracted follow-up pattern, cost, continuity of care

## Abstract

**Background:**

The use of multidisciplinary treatment programs in out-of-hospital healthcare is a new area of research. Little is known about the benefits of this method in the management of discharged patients undergoing cervical spondylosis surgery.

**Objective:**

This study aimed to explore the effect of a contracted-based, multidisciplinary follow-up plan in patients after cervical spondylosis surgery.

**Methods:**

This non-blinded non-randomized controlled study was conducted with 88 patients (44 in the intervention group, 44 in the control group). The clinical outcomes, including Neck Disability Index (NDI), pain score (VAS), Self-Efficacy for Managing Chronic Disease 6-item Scale (SECD-6), and 12-Item Short-Form Health Survey (SF-12) score were assessed at the time of discharge, 24–72 h, 1 month, and 3 months post-discharge. The complications, patient satisfaction, and economic indicators were assessed at the final follow-up (3 months).

**Results:**

Patients who received contracted follow-up showed greater improvement in neck dysfunction at 24–72 h, 1 month, and 3 months after discharge compared to those who received routine follow-up (*p* < 0.001). At 1 month after discharge, the intervention group exhibited better self-efficacy (*p* = 0.001) and quality of life (*p* < 0.001) than the control group, and these improvements lasted for 3 months. The intervention group reported lower pain scores at 24–72 h and 1 month (*p* = 0.008; *p* = 0.026) compared to the control group. The incidence of complications was significantly lower in the intervention group (11.4%) compared to the control group (40.9%). The total satisfaction score was significant difference between the two groups (*p* < 0.001). Additionally, the intervention group had lower direct medical costs (*p* < 0.001), direct non-medical costs (*p* = 0.035), and total costs (*p* = 0.04) compared to the control group. However, there was no statistically significant difference in indirect costs between the two groups (*p* = 0.59).

**Conclusion:**

A multidisciplinary contract follow-up plan has significant advantages regarding neck disability, self-efficacy, quality of life, postoperative complications, patient satisfaction, and direct costs compared with routine follow-up.

## Introduction

Cervical degenerative disc disease is a common condition that can cause myelopathy and radiculopathy. It has been reported that 3.8–17.6% of the population experience pain in the neck (1). These symptoms can significantly impact a patient’s work and quality of life. A report from the England provides the first cost-estimate to their society, an annual cost of £681.6 million per year (2). Non-surgical methods are typically used to manage pain and neurological symptoms in the cervical spine. However, if these treatments are unsuccessful, surgical interventions may be considered (3–5). Enhanced recovery after surgery (ERAS) is an interdisciplinary, multimodal approach aimed at improving postoperative outcomes. This approach employs evidence-based protocols in the care of surgical patients (6). Evidence regarding the use of ERAS protocols in spine surgery suggests that they have the potential to expedite recovery, minimize postoperative pain, and reduce hospital stay duration (7, 8). Despite the benefits of ERAS protocols, the short duration of hospitalization often prevents healthcare staff from adequately addressing patients’ questions about their postoperative condition. Studies have shown that patients undergoing cervical spondylosis surgery may experience complications such as heterotopic ossification, facet joint changes, adjacent segment degeneration, dysphagia, superficial wound infections, and others (4, 5). Furthermore, patients require professional nursing services and guidance after being discharged from the hospital. This is crucial for the effective and safe implementation of postoperative rehabilitation programs, including setting functional goals, traditional exercise therapy, and cognitive-behavioral strategies following cervical surgery (9).

Continuing care after discharge from the hospital is crucial for improving function and disability in patients who have undergone cervical spondylosis surgery. Multidisciplinary treatment programs that focus on biopsychosocial rehabilitation have shown promise in addressing both physical and mental health problems in patients with neck pain, and similar components may apply to the spine surgery population (10, 11). Implementing a family physician-contracted service can enhance the continuity and coordination of care, reduce inappropriate use of specialty services, and improve overall population health (12, 13). Many countries and regions, including England, Cuba, Australia, the United States, and Canada, have already implemented a family doctor system (14, 15). In China, previous studies have shown that the family doctor system has positive effects on improving health outcomes, such as lower hospitalization rates, reduced visits to the emergency department, and higher patient satisfaction levels among those with chronic diseases (16, 17). However, there are still many problems to be solved, such as family physician shortages, inadequate contract service rates, and the absence of supporting policies. Currently, the follow-up management of patients with cervical spondylosis is often carried out by a single healthcare professional, and most of the existing plans only focus on patients’ functional exercises, lacking comprehensive attention to their multidimensional health needs, such as psychological well-being, nutrition, pain management, and medication (18, 19). The evaluation of follow-up methods is often limited to a single outcome measure, without considering the cost-effectiveness of the follow-up plan.

Our study utilizes the continuity of care model proposed by Sarah et al. (20). According to this model, there are overlapping and hierarchical relationships among three dimensions of continuity of care: patient-provider relationship, communication, and collaboration. The model offers a framework for designing follow-up plans, aiming to prevent fragmented care and promote care continuity. The multidisciplinary follow-up pattern primarily consists of five components: Management of the multidisciplinary team, objectives, and content, settings, follow-up pathways, and patient experience. Our study, for the first time in China, introduced contract-based multidisciplinary team collaboration in the follow-up of patients after cervical spine surgery. Based on the continuity of care model, multidisciplinary teams provided effective guidance and interventions to patients in each dimension, aiming to enhance care continuity and assess its impact on patient care practices and outcomes.

This study aimed to investigate the short-to medium-term effects of a contracted-based, multidisciplinary follow-up pattern on patients who have undergone surgery for cervical spondylosis. The study specifically focused on comparing the effects of a multidisciplinary follow-up pattern with regular follow-up in terms of neck disability, pain, self-efficacy, health-related quality of life, complications, patient satisfaction, and economic indicators.

## Methods

This study was designed as a prospective, non-randomized controlled study with 3 months follow-up. The study adhered to the principles of the Helsinki Declaration (21) and the Transparent Reporting of Evaluations with Nonrandomized Designs (TREND) (22) statement regarding transparent reporting. Blinding was not applicable in this study. All patient data were used strictly for research purposes, and patient privacy was rigorously protected. Written informed consent was obtained from all patients or their guardians.

### Ethics approval

The study was approved by the Ethics Committee of West China Hospital, Sichuan University (No. 971; ref: 2021).

### Sample size calculation

Sample size calculation was performed using PASS 15 software with a two-sided significance level of *α* = 0.05 and statistical power of *β* = 0.2. Based on preliminary study results, the Neck Disability Index (NDI) was selected as the primary outcome measure with a standard deviation of 2 and a margin of error of 1.4. The sample size was estimated, and *n1* = *n2* = 35 was obtained. Considering a dropout rate of 20%, we estimated that a total of approximately 88 patients would be required (44 per group).

### Participants

A total of 88 patients diagnosed with confirmed cervical spondylosis were assigned to undergo cervical spondylosis surgery. The patients were discharged between June 2, 2021, and July 7, 2022. Inclusion criteria for the study were: (1) age ≥ 18 years; (2) eligibility for cervical spondylosis surgery; and (3) ability to provide informed consent, communicate effectively, and understand/read Chinese language. All discharged patients were required to meet uniform discharge criteria based on standard clinical care.

Patients with a history of previous cervical spine surgery, diagnosed dementia, blindness, or deafness, cervical malformation or significant instability, a history of severe cervical trauma, pregnancy, rheumatoid arthritis, malignancy, active infection, or other systemic diseases, as well as patients who are not recommended for follow-up, and those who faced difficulty in attending in-person visits for diagnostics or treatment or declined to participate, were excluded from the study.

### Study design

This study utilized convenience sampling as the sampling method. Patient recruitment was conducted prior to hospital admission. The head of the medical team explained the purpose and process of the program to patients who were about to undergo cervical spine surgery and invited them to participate in the study. Patients who agreed to participate were required to sign an informed consent form, confirming their voluntary participation in the research. Since the terms of the multidisciplinary follow-up contract needed to be personally confirmed and signed by the patients, blinding was not applicable in this study. Patients were non-randomly separated into two groups based on whether they signed a multidisciplinary team follow-up contract: patients who signed the contract were placed in the intervention group, while those who did not sign were placed in the control group. The sequential enrollment process started with the first eligible patient who signed the informed consent and continued until the last patient completed the 3-month follow-up study.

### Procedures

The study period was from June 2, 2021, when the first patient was enrolled, to July 7, 2022, when the last patient completed the follow-up. The study was conducted at the spinal surgery ward of West China Hospital, Sichuan University.

### Intervention group

This study utilized the continuity of care model proposed by Sarah et al. (20). The model suggests that there are overlapping and hierarchical relationships among the three dimensions of continuity of care: patient-provider relationship, communication, and collaboration. The theoretical dimensions mentioned involve the three types of continuity as identified by Haggerty et al. (23): relational continuity, informational continuity, and coordination continuity. Additionally, continuity is only required when there are changes in time and settings, which serves as the foundation for these three types of continuity. Our multidisciplinary follow-up pattern primarily consists of five components: Management of the multidisciplinary team, objectives, and content, settings, follow-up pathways, and patient experience. According to the TIDier checklist, we provided a detailed description of the intervention measures for the multidisciplinary follow-up pattern (Supplementary material 1).

### Control group

Patients in the control group received standard preoperative and postoperative nursing care following the established protocols for cervical spondylosis upon their admission to the hospital. One day before discharge, the responsible nurse and medical team leader provide the patient with pre-discharge health education, including postoperative skin and wound management, common discomfort symptoms after surgery, discharge instructions, postoperative rehabilitation exercise plan for cervical spondylosis, methods and duration of wearing a cervical collar, guidance on pain medication, hospital health consultation hotline, outpatient follow-up, and reexamination schedule, methods for appointment and registration, as well as distribution of postoperative health education materials. After discharge, the patient needs to make an appointment for postoperative follow-up visits for cervical spondylosis, at 1 month, 3 months, 6 months, and 1 year after the surgery. During the postoperative outpatient follow-up visits, the patient’s appointed physician will assess their health condition, provide medical advice based on the patient’s health needs, and offer corresponding health guidance.

### Data collection

The assessor (follow-up nurse), who had completed relevant professional courses, filled out a follow-up questionnaire. The assessor is aware of the distribution of patients in both groups. To ensure survey quality, a second researcher was invited to assist in the removal of invalid questionnaires. The primary outcome measure was neck pain-related disability, assessed using the NDI (0–100). Secondary outcome measures included the Visual Analog Scale (VAS), Self-Efficacy for Managing Chronic Disease 6-item Scale (SECD-6), and Study 12-Item Short-Form Health Survey (SF-12), which were used to assess neck pain, self-efficacy, and health-related quality of life. Additional measures included complications, patient satisfaction, and economic indicators. Patient characteristics, such as age, gender, marital status, qualifications, occupation, telephone number, length of stay, employment status, number of people living together, family income, body mass index (BMI), number of previous hospitalizations, admission patterns, cervical spondylosis classification, number of surgical segments, American Society of Anesthesiologists (ASA) physical status classification of I, II, or III, and convenience of medical treatment, were obtained from medical records and patient responses at baseline (the day of discharge). The NDI, VAS, SECD-6, and SF-12 were collected on the day of discharge, 24–72 h, 1 month, and 3 months after discharge. Patient satisfaction, complications, and economic indicators were recorded at 3 months after discharge. Data on the day of discharge for both groups of patients were collected in the ward. Data for the intervention group after discharge were collected through the Cervical Spondylosis Surgery Follow-up Platform, while data for the control group after discharge were collected either in the outpatient department or through telephone follow-up.

The NDI (24) is a 10-item questionnaire that measures the impact of neck symptoms on functional activities, with each item scored from 0 to 5, a higher score indicates a higher disability. The NDI covers pain, personal care, lifting, reading, headaches, concentration, work, driving, sleeping, and recreation. The NDI had acceptable responsiveness and construct validity to assess self-perceived disability (25).

The VAS (26) is a 100 mm horizontal line used to measure neck pain. The left end of the scale represents “no pain” and the right end represents the “most severe pain imaginable.” Patients mark the location that best represents their pain intensity based on their own condition and degree of pain.

Six items were included in the SECD-6 (27) to help assess how confident patients are in doing certain activities. The scale ranges from 1 (not at all confident) to 10 (totally confident). The score for the scale is the mean of the 6 items, and high scores indicate high self-efficacy.

The SF-12 (28), a measure of Health-related Quality of Life, is a shortened version of the SF-36 with 12 items, covering eight dimensions–general health (GH), physical functioning (PF), role-physical (RP), bodily pain (BP), vitality, social functioning (SF), role-emotional (RE), and mental health (MH). The eight dimensions can be consolidated into two summary scores using scoring algorithms, a physical component score (PCS) and a mental component score (MCS), ranging from 0 to 100, with higher scores representing a better health-related quality of life.

The follow-up satisfaction questionnaire consists of 10 items, covering satisfaction with the follow-up staff, health education, rehabilitation guidance, medical services, outpatient consultation, appointment registration process, auxiliary examination process, physical health, mental health, and costs related to cervical spondylosis after discharge. Responses are graded from 1 (not at all satisfied) to 5 (very satisfied), with higher scores representing higher satisfaction.

Economic indicators encompass direct medical costs, direct non-medical costs, indirect costs, and total costs. The estimation of direct medical costs and direct non-medical costs is conducted using the micro-costing method (29). These cost data are derived from hospital systems and patient interviews. Direct medical costs consist of post-discharge registration fees, medication expenses, treatment fees, examination fees, costs related to the treatment of complications, and other medical expenditures directly associated with the disease. Direct non-medical costs comprise transportation and accommodation expenses incurred by patients during medical visits. Total transportation costs are calculated based on patients’ reported round-trip transportation expenses and the number of visits made after discharge. Accommodation costs are estimated based on patients’ self-reported expenditures. Indirect costs pertain to income loss following cervical spine surgery and are estimated based on the patient’s lost work time post-discharge, converted into costs using daily wages as a basis. For employed individuals or those with fixed monthly incomes, indirect costs are represented by the total hours or days of productivity lost (seeking care and productivity time lost) multiplied by the average hourly or daily earnings. Total costs encompass both direct and indirect costs.

### Statistical analysis

Descriptive statistics, mean and standard deviation (SD) for normally distributed continuous variables, median and interquartile range (IQ) for non-normally distributed continuous variables, and absolute numbers and percentages for categorical variables were calculated to summarize the data. Since the data included repeated measurements and did not follow a normal distribution, we used Generalized Estimating Equations (GEE) to examine the effects of interventions on disability, pain intensity, self-efficacy, and health-related quality of life. In our analysis, we considered the intervention group (contract follow-up vs. routine follow-up), visit number (as a categorical variable), and the interaction between the intervention group and visit number as independent variables. We used robust standard errors and specified an exchangeable working correlation structure. We utilized all available time-point data. Differences between medians and their 95% confidence intervals were calculated using the Hodges-Lehmann estimator. For normally distributed categorical variables, we performed chi-square tests. For cases suitable for Fisher’s exact test, we used Fisher’s exact test. For normally distributed continuous variables, we conducted independent samples *t*-tests. For non-normally distributed continuous variables and ordered ordinal data, we used Mann–Whitney U tests. Results were considered significant at a 5% level of significance (*p* < 0.05). Data analysis was performed using licensed STATA 16 software, and graphs were generated using licensed GraphPad Prism 9.0.

## Results

**Figure 1:**
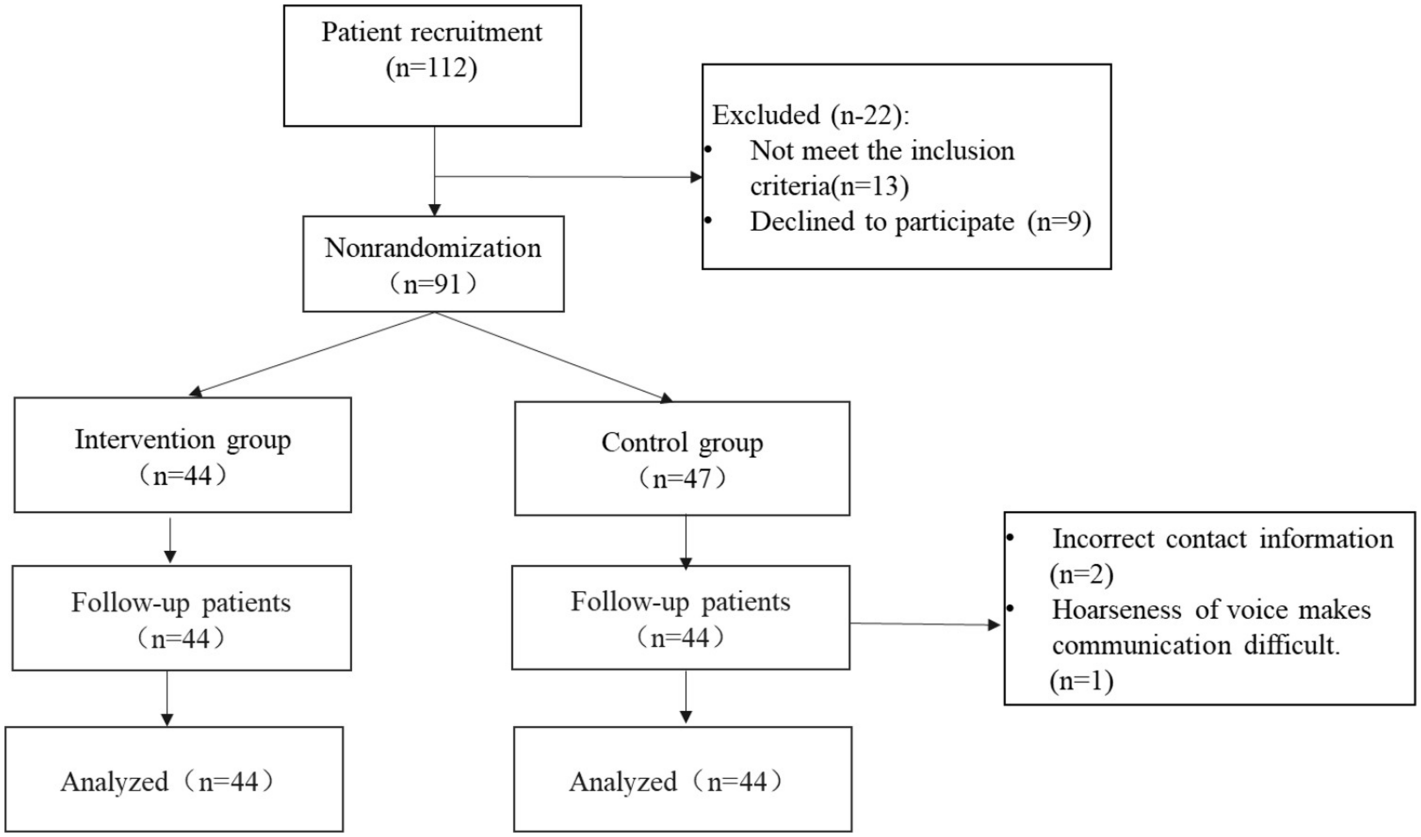
Study schematic panel.

The study commenced in June 2021, and the final follow-up was concluded in July 2022. A comprehensive three-month follow-up was conducted for all patients. Out of the initial pool of eligible patients, consisting of 91 individuals, only 44 agreed to participate in the intervention group, while 47 agreed to be part of the control group. Three patients from the control group withdrew from the study due to concerns regarding adherence to the protocol, as well as personal or other study-related issues. Ultimately, the study included a total of 88 patients who had undergone cervical spondylosis surgery, divided equally between the intervention and control groups, with 44 participants in each (Figure 1). The baseline characteristics of these 88 participants are presented in Table 1. Notably, there were no statistically significant differences observed between the two groups at baseline for all the variables that were examined, as anticipated.

**Table 1:** Baseline characteristics of patients.

Variables		Intervention group (*N* = 44)	Control group (*N* = 44)	*p*-value
Age Mean (SD)		51.64 (8.99)	52.43 (10.86)	0.7
Gender (female): number (%)		21 (47.7)	29 (65.9)	0.09
BMI (kg/m^2^): Mean (SD)		24.22 (2.62)	24.33 (2.94)	0.85
ASA:number (%)	Grade I	0 (0)	0 (0)	0.52
	Grade II	22 (50)	19 (43.2)	
	Grade III	22 (50)	25 (56.8)	
Surgical segments: number (%)	Single	18 (40.9)	13 (29.5)	0.27
	Multiple	26 (56.8)	31 (70.5)	
Length of stay Mean (SD)		5.41 (2.46)	5.94 (2.69)	0.2
Employment status: number (%)	Retired	13 (29.5)	16 (36.4)	0.49
	Working	31 (70.5)	28 (63.6)	
People living together: number (%)	Alone	0 (0)	2 (4.5)	0.49
	Gregarious	44 (100)	42 (95.5)	
Family income (per month)	<5,000	1 (2.3)	4 (9.1)	0.24
	5,001–10,000	14 (31.8)	17 (38.6)	
	>10,000	29 (65.9)	23 (52.3)	
Previous hospitalization Mean (SD)		24 (54.4)	28 (63.6)	0.39
Admission patterns: number (%)	Emergency	0 (0)	1 (2.3)	1
	Outpatient	44 (100)	43 (97.7)	
Cervical spondylosis classification: Mean (SD)	Radiculopathy	40 (90.9)	34 (77.3)	0.15
	Myelopathy	4 (9.1)	10 (22.7)	
Baseline NDI Mean (SD)		8.80 (6.71)	10.16 (7.60)	0.375
Baseline VAS Mean (SD)		2.01 (0.94)	2.15 (1.66)	0.627
Baseline SECD Mean (SD)		7.37 (1.09)	7.11 (1.34)	0.307
Baseline PCS M (IQR)		31.46 (13.13)	31.25 (18.75)	0.683
Baseline MCS M (IQR)		37.14 (23.24)	37.40 (9.92)	0.967

BMI, Body Mass Index; ASA, American Society of Anesthesiologists Physical Status Classification; SD, Standard Deviation; NDI, Neck Disability Index; VAS, visual analog scale; SCED-6, Self-Efficacy for Managing Chronic Disease 6-item Scale; SF-12, Study 12-Item Short-Form Health Survey; PHS, physical health score; MHS, physical health score.

### Primary outcome

**Figure 2:**
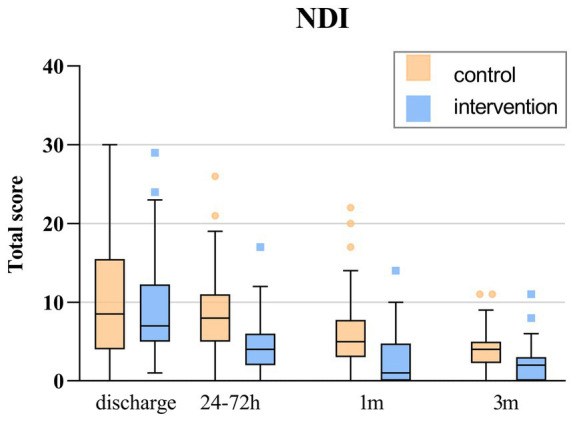
The total score of NDI over time. The box indicates P_25_ and P_75_. The horizontal line in the box represents the median of the NDI score. The error line represents the upper and lower limits of Tukey formula. Points outside the error line represent values for individual patients outside this range.

The comparison between groups revealed a significant difference in the NDI scores after discharge, specifically at 24 to 72 h, 1 month, and 3 months (*p* < 0.001), with the intervention group scoring notably lower than the control group. In addition, the intervention group exhibited sustained improvement in NDI scores at 24 to 72 h, 1 month, and 3 months after discharge according to within-group comparisons. However, there was no statistically significant difference in NDI scores among the control group during the first 24 to 72 h after discharge compared to initial baseline (Figure 2; Table 2).

**Table 2:** Summary of outcomes.

Parameter	Invention group M (IQR)^a^	Control group M (IQR)^a^	Between-group differences (95%CI)^b^	*p*- value^c^
NDI				
24–72 h	4 (4)***	8 (6)	-4 (−6, −2)	<0.001
1 m	1 (4.75)***	5 (4.75)**	-3 (−5, −2)	<0.001
3 m	2 (3)***	4 (2.75)***	-2 (−3, −1)	<0.001
VAS				
24–72 h	1 (1)***	1.55 (2.15)	−0.5 (−1, 0)	0.008
1 m	0.5 (1.2)***	1 (2)**	−0.3 (−1, 0)	0.026
3 m	0 (1)***	0 (1.38)***	0 (0, 0)	0.899
SECD-6				
24–72 h	8.75 (1.42)***	7.92 (2.08)***	0.67 (0.17, 1.33)	0.071
1 m	8.84 (1.67)***	8.5 (2.05)**	0.34 (0, 0.84)	0.001
3 m	9.17 (1.01)***	8.17 (1.96)**	1.17 (0.83, 1.67)	<0.001
PCS				
24–72 h	37.5 (18.75)***	37.5 (23.44)**	0 (0, 6.25)	0.362
1 m	87.5 (37.50)***	40.63 (42.19)***	31.25 (12.50, 50)	<0.001
3 m	81.25 (35.94)***	55.63 (56.25)***	25 (6.25, 31.25)	<0.001
MCS				
24–72 h	19.74 (47.37)	10.53 (10.53)	8.16 (3.95, 19.74)	0.949
1 m	98.68 (36.18)***	20.39 (76.71)*	35.53 (5.26, 60.53)	0.001
3 m	94.74 (23.68)***	60.53 (78.95)***	25 (5.26, 46.05)	<0.001

NDI, Neck Disability Index; VAS, visual analog scale; SCED-6, Self-Efficacy for Managing Chronic Disease 6-item Scale; SF-12, Study 12-Item Short-Form Health Survey; PCS, physical component score; MCS, mental component score.

^a^*, **, and *** indicated within-group differences at the *p* < 0.05, 0.01, and 0.001 values, respectively. For within-group comparisons, all comparisons were made relative to the scores at discharge.

^b^The median difference and 95% confidence interval (CI) for between-group comparisons were calculated using the Hodges-Lehmann estimator.

^c^Indicated the value of *p* for between-group comparisons.

### Secondary outcome

In this study, we observed that patients in intervention group had significant improvements in various secondary outcome measures, including SECD-6, VAS, PCS, and MCS, at the 1-month mark (*p* < 0.05, Table 2). Furthermore, at the 3-month mark, the intervention group exhibited a greater increase in SECD-6, PCS, and MCS scores compared to the control group, demonstrating statistical significance (*p* < 0.001). However, no significant differences were observed between the two groups within the 24-to-72-h period. Additionally, the VAS scores showed a statistically significant difference between the two groups at both the 24–72 h and 1-month time points (*p* < 0.05).

### Patient satisfaction

The findings demonstrate that the intervention group (mean 42.52, SD 8.2) is significantly more effective in meeting the needs of patients compared to control group (mean 41.56, SD 7.34) (*p* < 0.001, Table 3). Moreover, when assessing various aspects of the follow-up satisfaction questionnaire, patients in the intervention group consistently expressed significantly higher levels of satisfaction (*p* < 0.001, Table 3).

**Table 3:** Patient follow-up satisfaction.

Variables	Intervention group/Mean (SD)	Control group/Mean (SD)	Fisher	*p*-value
Satisfaction with follow-up staff	4.2 (0.90)	4.2 (0.76)	38.05	<0.001
Satisfaction with health education	4.16 (0.86)	4.18 (0.84)	34.87	<0.001
Satisfaction with rehabilitation guidance	4.23 (0.91)	4.2 (0.85)	33.63	<0.001
Satisfaction with medical services	4.3 (0.79)	4.43 (0.7)	48.71	<0.001
Satisfaction with outpatient consultation	4.25 (0.86)	4.18 (1.02)	49.57	<0.001
Satisfaction with registration	4.18 (0.99)	3.75 (0.94)	32.83	<0.001
Satisfaction with the auxiliary examination	4.2 (0.95)	3.98 (0.9)	29.4	<0.001
Satisfaction with physical health	4.27 (0.85)	4.32 (0.88)	31.7	<0.001
Satisfaction with mental health	4.36 (0.92)	4.16 (0.94)	35.32	<0.001
Satisfaction with the medical costs after discharge	4.36 (0.87)	4.16 (0.96)	32.29	<0.001
Patient overall satisfaction	42.52 (8.2)	41.56 (7.34)	43.46	<0.001

### Complications

During the study, a total of 11.4% of patients in the intervention group experienced complications, while 40.9% were in the control group (*p* < 0.01; Supplementary Table S1). Importantly, it is worth noting that no patients discontinued the study due to complications. When patients experience complications, they have been duly reported to the ethics committee. These complications are recognized as being caused by the patient’s underlying condition, rather than the intervention measures.

### Post-discharge costs

**Figure 3:**
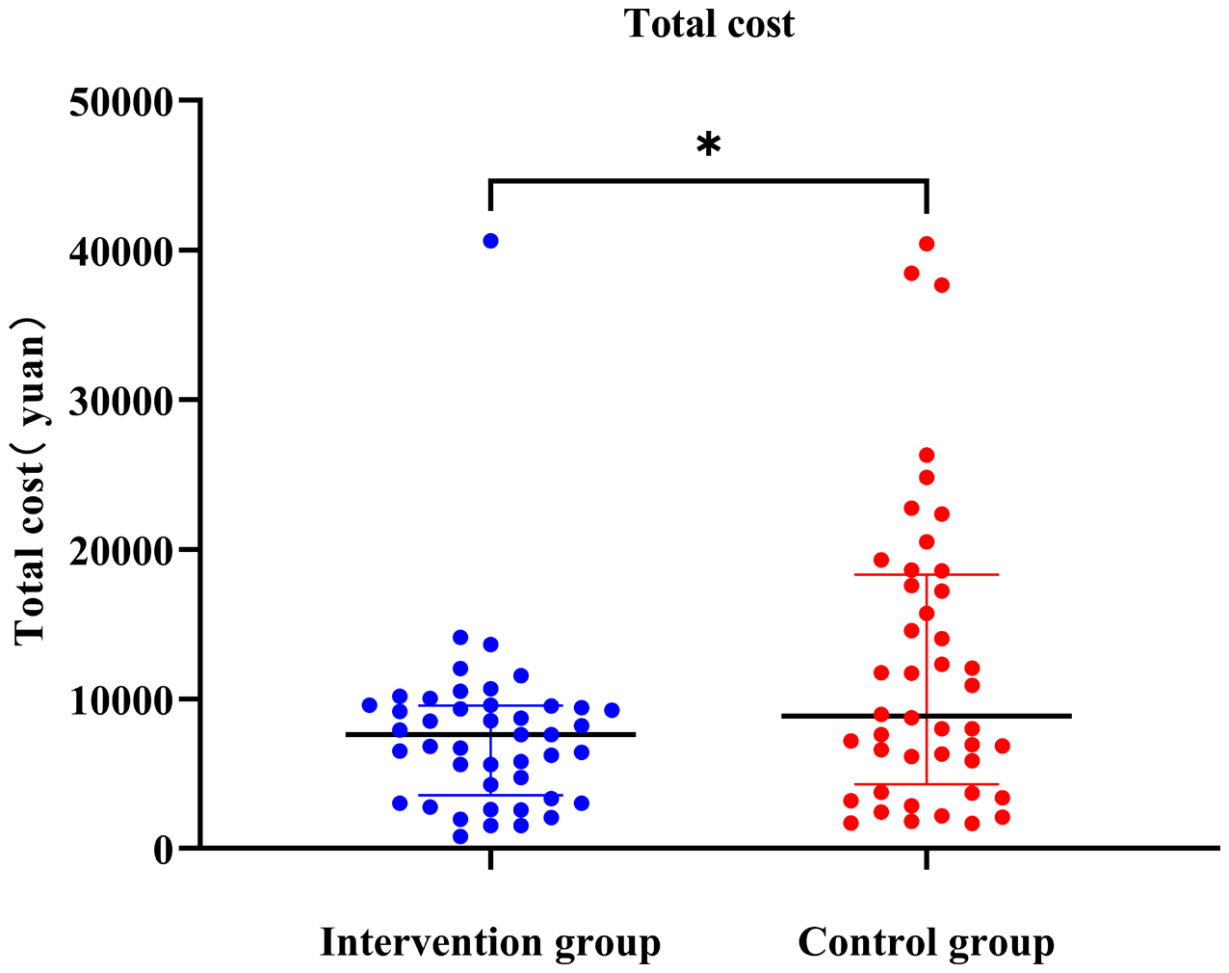
The total cost over time. *indicated within-group differences at the *p* < 0.05.

The findings of this study revealed significant differences in direct medical costs between the intervention group and the control group. The median direct medical cost in the intervention group was 1821.2 yuan, whereas it was 2421.7 yuan in the control group (*p* < 0.001; Supplementary Table S2; Figure 2). Furthermore, notable distinctions were observed in the median direct non-medical costs. The intervention group had a median direct non-medical cost of 110 yuan, whereas the control group had a median cost of 270 yuan (*p* = 0.035). Regarding indirect costs, no significant differences were found between the intervention group (median cost of RMB 4750) and the control group (median cost of RMB 5000) (*p* = 0.59). Moreover, the total cost of the intervention was significantly lower in the intervention group (7621.2 yuan) compared to the control group (8725.2 yuan) (*p* = 0.04) (Figure 3).

## Discussion

The main finding of this study indicates significant improvements in neck disability, self-efficacy, quality of life, post-operative complications among patients who received contract-based follow-up, compared to routine follow-up. Contract-based follow-up demonstrated higher levels of patient satisfaction and lower total costs after discharge.

Postoperative outcomes such as pulmonary, postoperative hematoma, and dysphagia have been demonstrated as complications of cervical spine surgery, and can also cause poorer patient prognosis after surgery (30). Complications of cervical spine surgery can arise after discharge. Without professional guidance, it can be very dangerous. Continuing care extends hospital care into daily life and maintains a connection between individual patients and the care team, with the primary goal of actively involving patients in their recovery process (31). In our study, we introduced a pioneering model of contracted continuous follow-up, utilizing a multidisciplinary team. Various medical experts from different disciplines fully utilize their professional skills to achieve complementary backgrounds and multiple layers of safety. This approach addressed the specific self-care needs of post-discharge patients, improving their quality of life and adherence to medical recommendations. Personalized services were provided under the contracted continuous follow-up model tailored to each patient’s requirements.

Compared to the control group, the intervention group showed potential improvements in neck dysfunction and pain, which may be attributed to the professional rehabilitation provided by doctors and better compliance from patients. Previous evidence suggests that proper exercise methods can contribute to reducing neck pain and disability in patients with cervical spondylosis (32). Some studies suggest that structured postoperative physical therapy may bring benefits to patients undergoing surgical treatment for cervical disc disease, compared to standard care methods (33, 34). Regular exercise may promote pain relief by reducing NMDA receptors phosphorylation and decreasing serotonin transporter expression (35). However, it has been shown by Wibault et al. (19) that the potential for further improvement in NDI and VAS scores through postoperative physical therapy may be limited, as these measures reflect immediate postoperative outcomes. Interestingly, our study also revealed no statistically significant difference in VAS scores between the two groups at 3 months after discharge. This lack of significance may be due to over 75% of patients having the lowest possible VAS score (0), indicating a floor effect. Consequently, there might be some debate surrounding the responsiveness of VAS as a tool for detecting pain changes during the postoperative period of cervical spondylosis. Therefore, it is essential to extend the follow-up period in future studies to observe the long-term effects of the contractual follow-up model on outcomes such as NDI and VAS.

Patients in the intervention group showed a higher sense of self-efficacy than the control group at 1 and 3 months after discharge. However, no significant difference was observed between the two groups within the first 24–72 h post-discharge, possibly due to the short-term nature of the intervention measures. The study highlights the importance of a multidisciplinary follow-up team that provides specialized pain neuroscience education, functional exercises, and psychological counseling to improve patients’ self-efficacy. Previous studies have demonstrated that comprehensive treatment including pain neuroscience education and functional exercises can help alleviate pain and disability in patients and enhance their self-efficacy (36). According to social cognitive theory, self-efficacy is critical to behavioral change, as individuals who believe in their thoughtful and deliberate actions are more likely to implement action plans. Higher self-efficacy can also reduce patient anxiety and self-doubt (37). Future studies should investigate more effective self-efficacy intervention methods that can be applied during post-discharge follow-up to improve patients’ self-efficacy and facilitate their recovery. In the first and third months after discharge, the intervention group showed significantly better quality of life compared to the control group. This may be attributed to the targeted intervention provided by a multidisciplinary team, such as psychologists and specialized nurses. Studies have demonstrated that tools like SF-36 and SF-12 can effectively assess patients’ quality of life (38). It is worth noting that a considerable proportion of cervical spine disease patients also suffer from psychological disorders. It has been reported that more than 30% of cervical spine disease patients have depression or anxiety (39). Previous research indicates that higher SF-MCS scores before surgery are associated with better post-operative quality of life, improved psychological well-being, and higher patient satisfaction (40). Therefore, timely psychological interventions before surgery are crucial. Looking ahead, expanding the scope of contracted follow-up services to include the pre-surgery stage can achieve continuous management throughout the entire process for cervical spine surgery patients, promoting their preoperative recovery.

Our study demonstrates that contracted follow-up management can reduce the incidence of complications at 3 months after cervical surgery. Early postoperative dysphagia and neurological complications were the most common complications in both groups. This may be attributed to the comprehensive care and specialized guidance provided by a multidisciplinary team, which effectively mitigated the risk of developing complications. Previous studies have reported a wide range of dysphagia incidence following cervical spine surgery, ranging from 17.5 to 71% (41). However, the exact causes of postoperative dysphagia in this context remain unclear. Factors such as the type of surgery, including multilevel procedures (particularly involving C4-5 and C5-6), age, smoking status, operative duration, and body mass index, have been identified as closely associated with the occurrence of early dysphagia (42, 43). Consequently, future research may benefit from conducting subgroup analyses to further elucidate the effects of contracted follow-up management, with a focus on specific surgical segments and operative duration. Our study indicated that the implementation of a contracted follow-up program, carried out by a multidisciplinary team, can greatly enhance patient satisfaction following cervical spine surgery. In this study, we established a dedicated postoperative follow-up management center designed specifically for cervical spine diseases. This center offers a range of services including online consultations, free appointments, physical examinations, test result interpretations, imaging analyses, and even convenient expert outpatient services available during the night. Additionally, the utilization of an intelligent electronic follow-up platform assists in the seamless and continuous storage of patients’ health information. In recent years, the practice of prehabilitation has started gaining traction within the field of orthopedics. However, there remains a lack of consensus regarding its ability to expedite patient recovery (44, 45). In the future, standard nursing procedures for preoperative rehabilitation should be explored for patients undergoing cervical spine surgery.

The intervention group of patients showed lower total costs, direct medical costs, and direct non-medical costs compared to the control group. Complications following cervical spine surgery can have a negative impact on postoperative patient outcomes (30). Postoperative complications related to cervical spine conditions can still occur after discharge, posing risks to patients and increasing healthcare expenses without professional guidance. A multidisciplinary team provides personalized services to patients when necessary, such as in-hospital referrals and emergency admissions, ensuring patient safety and reducing the occurrence of complications outside the hospital while also lowering healthcare costs. However, our study showed no significant difference in indirect costs between the two groups, which may be attributed to the relatively short study duration and potential recall bias in obtaining indirect cost data through patient interviews. Further research is required to investigate the long-term effectiveness and economic benefits of implementing a contracted follow-up model in spinal surgery.

### Limitation

There are several limitations associated with this non-randomized control study design. Firstly, due to the requirement of voluntary patient consent in contractual terms, a randomized study design and blinding was not feasible. Non-randomized controlled designs may suffer from selection bias, raising doubts about the reliability and validity of study findings. To mitigate potential biases arising from non-randomization, this study rigorously enforced inclusion criteria for participants and ensured that both groups of patients originated from the same medical group. The lack of blinding may cause researchers to exhibit heightened concern for the patients, potentially impacting the objectivity of the observed outcomes. Secondly, our follow-up period was relatively short, lasting only 3 months after discharge. A short follow-up time may result in insufficient assessment of patients’ post-discharge conditions and intervention effects. Future studies should incorporate long-term follow-ups to ascertain the long-lasting benefits of multidisciplinary contractual continuing care. Thirdly, this study was conducted at a single center, and the findings exclusively reflect the performance of that specific center. To enhance external validity, future research endeavors could encompass various geographical regions and diverse tiers of hospitals. Finally, our study protocol has not been previously published, which may have an impact on the credibility and transparency of the study. However, we have included a detailed research protocol (Supplementary material 1) in this study to facilitate reproducibility by other researchers.

## Conclusion

Compared with the routine follow-up plan, the multidisciplinary contracted follow-up plan demonstrates significant benefits for postoperative cervical dysfunction, self-efficacy, quality of life, complications, patient satisfaction, and direct costs in patients undergoing cervical spine surgery.

## Data availability statement

The data analyzed in this study is subject to the following licenses/restrictions: the data that support the outcomes of this paper are available from NN, upon reasonable request. Requests to access these datasets should be directed to NN, ningninggk@163.com.

## Ethics statement

The studies involving humans were approved by Ethics Committee of West China Hospital, Sichuan University. The studies were conducted in accordance with the local legislation and institutional requirements. The participants provided their written informed consent to participate in this study. Written informed consent was obtained from the individual(s) for the publication of any potentially identifiable images or data included in this article.

## Author contributions

ZF: Writing – original draft, Data curation. YX: Writing – review & editing, Methodology, Investigation. LP: Writing – review & editing, Supervision, Formal analysis. MG: Supervision, Software, Writing – review & editing. JC: Writing – original draft, Project administration, Data curation. NN: Writing – review & editing, Resources, Conceptualization.
