# Supplementary material for: Assessing multidisciplinary follow-up pattern efficiency and cost in follow-up care for patients in cervical spondylosis surgery: a non-randomized controlled study
Source: Front Med (Lausanne). 2024 Apr 3;11:1354483. doi: 10.3389/fmed.2024.1354483 (PMC11022215; doi:10.3389/fmed.2024.1354483)
Supplement: Supplementary file 1 [file Data_Sheet_1.DOCX]

**Table S1. Comparison of Adverse Events**

| Variables | Intervention Group/No. (%) | Control  Group/ No. (%) | Z | *P* value |
| --- | --- | --- | --- | --- |
| Dysphagia | 1(2.3) | 6(13.6) | -2.96 | <0.01 |
| Neurological complications | 2(4.5) | 9(20.5) |  |  |
| Hematoma | 0 | 1(2.3) |  |  |
| Recurrent laryngeal nerve paralysis | 1(2.3) | 1(2.3) |  |  |
| Adjacent cervical disc degeneration | 1(2.3) | 1(2.3) |  |  |
