# Supplementary material for: Assessing multidisciplinary follow-up pattern efficiency and cost in follow-up care for patients in cervical spondylosis surgery: a non-randomized controlled study
Source: Front Med (Lausanne). 2024 Apr 3;11:1354483. doi: 10.3389/fmed.2024.1354483 (PMC11022215; doi:10.3389/fmed.2024.1354483)
Supplement: Supplementary file 2 [file Data_Sheet_2.DOCX]

**Table S2. Comparison of Post-discharge costs**

| Variables | Intervention Group (M/IQR) | Control  Group (M/IQR) | Z | *P* value |
| --- | --- | --- | --- | --- |
| Registration cost | 0(0) | 84.0(80) | 54.21 | <0.001 |
| Medication cost | 0(500) | 500.0(880) | 8.73 | 0.003 |
| Laboratory test fee | 1521.2(0) | 1521.2(0) | 1.19 | 0.28 |
| Treatment fee | 0(0) | 0(240) | 5.38 | 0.02 |
| Cost of treating complications | 0(0) | 0(700) | 9.16 | 0.002 |
| Other directly related medical expenses | 0(0) | 0(260) | 3.97 | 0.05 |
| Total direct medical expenses | 1821.2(820) | 2421.7(3828.9) | -3.5 | <0.001 |
| Transportation expenses | 100.0(240) | 200.0(360) | 3.58 | 0.06 |
| Accommodation expenses | 0(0) | 0(180) | 2.91 | 0.09 |
| Indirect costs up to the present follow-up | 100.0(10,350) | 280.0(60,735) | 4.41 | 0.04 |
| Days of productivity loss | 10.0 (24) | 16.7(30) | -0.25 | 0.81 |
| Average daily income loss | 150.0（260） | 166.7(333.33) | -0.43 | 0.67 |
| Indirect costs | 4750.0 (7900) | 5000.0(10000) | -0.47 | 0.59 |
| Total costs | 7621.2 （6190） | 8725.2（13806.6） | -2.1 | 0.04 |
