# Supplementary material for: Assessing multidisciplinary follow-up pattern efficiency and cost in follow-up care for patients in cervical spondylosis surgery: a non-randomized controlled study
Source: Front Med (Lausanne). 2024 Apr 3;11:1354483. doi: 10.3389/fmed.2024.1354483 (PMC11022215; doi:10.3389/fmed.2024.1354483)
Supplement: Supplementary file 3 [file Data_Sheet_3.DOCX]

***Supplement 1***

***Item 1: Brief name***

Multidisciplinary follow-up pattern

***Item 2: rationale, theory or goal of the elements essential to the intervention***

In order to implement ERAS clinical pathways effectively, it is important to integrate various perioperative interventions and establish a patient-centered multidisciplinary team collaboration that incorporates modern technology and theory to expedite patient recovery. Patients undergoing cervical spine surgery may still encounter physiological and psychological challenges post-surgery, and their need for professional follow-up after discharge is significant. A single post-discharge follow-up may not be sufficient to address all of the patient's health concerns. Therefore, within the context of ERAS, multidisciplinary team collaboration in post-discharge follow-up can serve as a crucial approach to promote patient rehabilitation outside of the hospital setting. The concept of multidisciplinary team collaboration was initially adopted by the Mayo Clinic as a medical nursing model, where a team consisting of doctors and other healthcare professionals provided comprehensive medical and nursing care to patients. Since then, multidisciplinary team collaboration has become an integral aspect of modern medicine (1). Multidisciplinary practice refers to a team of healthcare professionals from different disciplines working together with the common objective of delivering comprehensive care services. In a multidisciplinary practice, each member assumes an independent role while respecting the boundaries of their respective disciplines. Through experience, each member can expand their scope of practice and take on elements of other roles to effectively manage workloads. For instance, nurses can conduct clinical assessments while physical therapists can offer treatment advice on behalf of the entire team (2). Studies have demonstrated that multidisciplinary teams can enhance the prognosis and survival rates of cancer patients (3). Moreover, in the field of orthopedics, multidisciplinary teams have proven effective in treating multiple traumas (4), hip fractures (5), and joint replacements (6). Multidisciplinary treatment plans that focus on biopsychosocial rehabilitation have also shown promise in addressing the physical and mental health issues of patients with neck pain, and these findings are likely applicable to patients undergoing spinal surgery (7, 8). However, there is limited research on the application of multidisciplinary teams in post-discharge follow-up after cervical spine surgery, with most studies primarily concentrated on patient assessment, diagnosis, treatment, and in-hospital nursing care (9-11).

The key focus of the contract-based follow-up pattern is to provide scheduled follow-up appointments to ensure continuous and convenient care. This approach draws inspiration from the concept of signing contracts with family doctors. Family doctor contract services are essentially an extension and advancement of community health services that prioritize informed consent, voluntary contract agreements, and standardized care. By entering into service contracts with residents, proactive, continuous, and comprehensive health management models are delivered (12). Research indicates that contract services can improve care continuity and coordination, lower healthcare resource waste, decrease emergency department visits, and enhance population health (13, 14). To date, over 50 countries and regions, including the United Kingdom, Cuba, Australia, the United States, and Canada, have implemented family doctor contract systems (15, 16). While the professionals involved in family doctor services may vary slightly among countries, their qualifications are strictly regulated. Previous studies have demonstrated that the family doctor system positively impacts health outcomes, such as reducing hospitalization rates, decreasing emergency department visits, and increasing satisfaction among patients with chronic diseases(17). Currently, family doctor contract services have been implemented in various regions across China, including Shanghai, Zhejiang, urban, suburban, and rural areas (12, 18-20). However, several challenges remain, including a shortage of family doctors, low contract service rates, and limited supporting policies. Additionally, the majority of family doctors in China consist of general practitioners, physician assistants, or rural doctors (19), who may lack experience in providing specialized nursing and rehabilitation treatments for patients undergoing complex procedures like cervical spine surgery. As a result, they may struggle to meet the demands for accelerated patient recovery. Therefore, a specialized and multidisciplinary team-based contract service model may offer a novel outpatient follow-up approach that can effectively address the complex and personalized needs of post-surgery patients with cervical spine disorders.

Continuity of care refers to the integration of hospital nursing services into daily care, ensuring a consistent relationship between individual patients and the nursing team, with the ultimate goal of promoting patient self-initiated care (21). In this study, we utilized the continuity of care model proposed by Sarah et al. (22) as a theoretical framework to develop a new follow-up service model for postoperative cervical spine patients. Drawing inspiration from contracted family physician services, we established a contract-based system that provides professional, comprehensive, continuous, and convenient follow-up services for postoperative cervical spine patients through multidisciplinary team collaboration. This model caters to the diverse and comprehensive follow-up needs of patients and ensures high-quality care throughout the entire recovery process.

***Item 3: Materials used in the intervention***

Multidisciplinary contract-based follow-up involves the comprehensive management of patients through follow-up services. To facilitate this process, the Cervical Spondylosis Surgery Follow-up Platform and paper-based health information files have been meticulously developed. The Cervical Spondylosis Surgery Follow-up Platform comprises various sections such as detailed medical history, appointment scheduling (including expert consultations and examinations), test results, health assessment, and internal referrals. This platform is accessible to both follow-up doctors and nurses, enabling efficient coordination of care. The paper-based health information files encompass three essential components: patient demographic details, telephone follow-up forms, and specialized assessment scales. Dedicated follow-up nurses conduct thorough telephone follow-ups, encompassing assessments of general well-being, guidance on neck function exercises, instructions on proper usage of neck braces, medication guidelines, psychological health education, and effective management of any potential complications. Furthermore, upon discharge, patients are provided with follow-up cards containing crucial information regarding the follow-up location, date, and important care instructions.

***Item 4: Procedures, activities and/or processes used in the intervention***

Our multidisciplinary follow-up pattern primarily consists of five components: Management of the multidisciplinary team, objectives, and content, settings, follow-up pathways, and patient experience.

*Management of the multidisciplinary team:* To ensure the continuity and exchange of information, a multidisciplinary follow-up team led by the head nurse and medical group leader was established. The team comprises follow-up physicians, follow-up nurses, wound care specialists, pain management nurses, psychologists, and nutrition specialists who collaborate with patients' families in making decisions and resolving their health issues accurately. Both patients and their families participate throughout the follow-up process. Each patient is assigned a dedicated follow-up nurse and physician, ensuring consistent care, and all follow-up physicians possess experience in the specific surgical procedures undergone by the patients.

*Objectives and content:* (a) Comprehensive Care in the Entire Disease Course: The multidisciplinary contracted follow-up program includes medical history collection, health risk assessment, psychological evaluation, recording and interpretation of examination results, disease progression monitoring, and continuous disease care services, all centered around the patient's health. (b) Follow-up Management: Personalized and standardized health management services are provided. The follow-up doctors and nurses are familiar with the patient's surgical and disease conditions, enabling smooth communication between medical professionals and patients to effectively address health issues in real time. (c) Medical Planning: The multidisciplinary contracted follow-up team provides patients with medical planning services related to cervical spine surgery, including guidance on physical examinations, consultation advice, and expert consultations on imaging, medication, and rehabilitation training. (d) In-Hospital Referrals: If patients develop other systemic diseases or severe complications during the follow-up process, in-hospital referral services will be provided to facilitate prompt access to comprehensive healthcare. Additionally, in case of urgent medical conditions, coordination with hospital emergency departments can be arranged, providing expedited appointments and facilitating urgent hospital admissions through a streamlined process to effectively handle any complications occurring outside the hospital. (e) Convenient Medical Care: We streamline the routine follow-up and review process for patients after discharge. Follow-up nurses proactively schedule appointments for examinations and consultations with specialists, allowing patients to undergo specialized examinations and receive results and treatments on the same day as their follow-up visit.

*Follow-up settings*: The program mainly includes fixed-location clinics and internet-based follow-up. Fixed-location clinics have stable schedules and convenient locations for patients to seek medical care. For patients visiting the follow-up clinic for the first time, follow-up nurses will provide pre-visit instructions on using the hospital's navigation app. When necessary, the follow-up team will guide patients through examination and medication processes at the clinic. Internet-based follow-up is primarily conducted through WeChat and the hospital's app. Follow-up nurses contact patients via WeChat to provide health guidance, answer questions, and handle emergencies. Patients can also engage in text-based consultations through the hospital's app. During the internet-based follow-up process, follow-up nurses should respect patients' opinions and ensure the protection of their privacy.

*Follow-up pathways:* Before admission, the medical team leader provided detailed information about the "Contracted Follow-up Plan" to the patients and ensured that all patients signed the follow-up contract with informed consent. On the one hand, follow-up nurses created health information files for each contracted patient. On the other hand, contracted patients are automatically enrolled in the “Cervical Spondylosis Surgery Follow-up Platform”, which connects all patient information since their admission and incorporates specialized assessment scales to facilitate continuous care. Patients can add a "follow-up WeChat" to contact our multidisciplinary team for postoperative emergencies and health consultations. For elderly patients who are unable to use smartphones, their emergency contacts (or legal representatives) will handle the registration process. On the day of admission, patients are required to complete the Patient Basic Information Form in the Health Management File. A follow-up nurse will accompany the cervical spine surgery medical team to check on the patient's condition every morning. For patients with special conditions, follow-up records will be made in the follow-up health management file for post-discharge follow-up purposes. Based on the patient's actual situation, a multidisciplinary team will discuss and develop an outpatient follow-up plan. One day before the patient's discharge, the responsible nurse will provide pre-discharge health education together with the follow-up physician and provide the postoperative health education manual. The follow-up nurse will make an appointment with the patient for the first post-discharge outpatient follow-up visit and record the information on the follow-up WeChat account while issuing a follow-up card (including the follow-up location, time, and precautions).

Due to the serious situation of the COVID-19 epidemic, a combined approach of outpatient follow-up, telephone follow-up, and internet follow-up has been adopted for the post-discharge follow-up plan.

Outpatient follow-up: According to expert consensus, patients should have follow-up visits at the outpatient clinic at 1 month, 3 months after surgery (23-25). In the week before each follow-up time point, the patient's appointment for examinations and follow-up content should be arranged in consultation with the follow-up physician. In addition to functional and pain assessments, it should also include guidance on wearing neck braces, correct use of analgesics, and functional exercises. Each member of the multidisciplinary team is responsible for their respective tasks in the outpatient follow-up. If the patient also suffers from other chronic diseases, appointments with specialists from other departments can be arranged, and communication with members of the multidisciplinary follow-up team is conducted to promptly address the patient's issues and ensure real-time information sharing. In case of special circumstances, the follow-up nurse can adjust the follow-up time based on the follow-up physician's advice and inform the patient of the next follow-up time and post-discharge precautions. Family members should be involved throughout the outpatient follow-up process.

Telephone follow-up: After discharge, nurses will conduct telephone follow-up at 24-72 hours, 2 weeks, 3 weeks, respectively. The follow-up content includes a general assessment of the patient's condition, guidance on neck function exercise, neck brace wearing guidance, medication guidance, psychological health education, management of complications, etc. For problems and difficulties that need to be resolved for patients, nurses should make records and communicate with other members of the multidisciplinary follow-up team to provide timely feedback and solutions. At the same time, relevant information should be entered into the “Cervical Spondylosis Surgery Follow-up Platform” and health management files to ensure information continuity.

Internet-based follow-up service: The official WeChat public account and hospital APP have a "follow-up service" section. After discharge, patients can use this service for consultation on cervical spine surgery, continuous medical advice, and online prescriptions. At the same time, if patients encounter any questions related to their cervical spine surgery while at home, they can contact follow-up nurses through the follow-up WeChat account. The follow-up nurses will activate the corresponding follow-up procedures based on the patient's condition and record the information in the electronic follow-up system and health management records to ensure information continuity. For those patients for whom remote resolution of specific problems is not possible, dynamic coordination should be made to refer them to the corresponding medical team's follow-up outpatient clinic. In areas where the COVID-19 pandemic is severe and it is not possible to visit the outpatient clinic for follow-up, the follow-up nurse should guide patients in functional exercises through methods such as video instruction. For patients with psychological issues, psychologist will provide remote psychological interventions. For patients with wound infection or abnormal conditions, wound care nurses will provide guidance and treatment. Under the guidance of the follow-up physician, patients can visit local hospitals for relevant imaging examinations before the scheduled follow-up appointment and upload the results to the follow-up WeChat account. The follow-up physician will interpret the results and provide health guidance. Specifically, if a contracted patient encounters an emergency after being discharged, a "green channel" will be opened immediately to assist with readmission or provide consultation through an online hospital. Additionally, during the follow-up period, each contracted patient has opportunity to be referred to other chronic disease departments for treatment of non-cervical related diseases within the validity period of the agreement.

*Patient experience*: To improve patients' healthcare experience and achieve integrated management among healthcare providers, patients, and caregivers, several measures have been taken during the follow-up process. Firstly, regular training and education sessions are organized for multidisciplinary follow-up teams to ensure that patients receive the latest medical advice and treatment plans. Secondly, during outpatient follow-ups, the follow-up team takes the initiative to ask for patients' opinions and feedback to understand their satisfaction with the follow-up services. By assessing patient feedback, problems and areas for improvement can be identified promptly, thereby enhancing the quality of follow-up services. Finally, an open platform is established for collecting patients' follow-up opinions. This platform allows patients to freely provide suggestions or feedback. Through this platform, valuable insights and needs of patients can be effectively collected, enabling appropriate improvements and adjustments based on their opinions.

***Item 5: Description of the expertise, background and specific training given to intervention***

***provider***

*All members of the follow-up team must meet the following criteria*: (a) hold a bachelor's degree or higher in their respective field; (b) possess a minimum of 10 years of relevant professional work experience; (c) have the necessary professional certification and licensure. Additionally, follow-up nurses should also have excellent communication skills and the ability to adapt to unexpected situations. They should be capable of applying clinical thinking to address health-related problems or difficulties encountered by the patients.

*Training for members of the multidisciplinary contract follow-up team*: It is essential to regularly schedule specialized training lectures, both domestic and international, for follow-up doctors, follow-up nurses, wound care specialists, pain management nurses, psychotherapists, and nutrition specialists. This training aims to enhance their professional skills and ensure the quality of follow-up care. Each team member should attend the corresponding training annually. Additionally, regular monthly multidisciplinary follow-up team meetings should be organized by the head nurse. Attendance is mandatory for all team members to promote real-time sharing and tracking of patients' latest health status, address any issues encountered during follow-up, and make timely adjustments to the individualized follow-up plans based on patient needs.

***Item 6: Mode of delivery***

The multidisciplinary contract-based follow-up plan is implemented through a comprehensive approach that combines outpatient follow-up, telephone follow-up, and internet-based follow-up.

***Item 7: Type(s) of location(s) where the intervention occurred, including any necessary infrastructure***

This intervention measure is implemented at the Department of Spine Surgery, West China Hospital of Sichuan University, encompassing both general inpatient wards and follow-up outpatient clinics.

***Item 8: Number of times the intervention was delivered and over what period of time including the number of sessions, their schedule, and their duration, intensity or dose***

The patients in the intervention group underwent a three-month multidisciplinary follow-up program.

***Item 9: Tailoring of the intervention***

The intervention measures employed in this study were personalized. Throughout the follow-up process, the follow-up strategies were dynamically adjusted based on the patients' condition to meet their unique healthcare needs.

***Item 10: Modifications of the intervention during the study***

No amendments were made to the intervention during the study.

***Item 11: How adherence or fidelity was assessed***

One day prior to discharge, we collected two or more contact methods from each patient to ensure continuous communication between the patient and the multidisciplinary contracted team after discharge.

***Item 12: Actual adherence or fidelity***

All 44 patients (100%) successfully completed the three-month multidisciplinary contracted follow-up program, with no dropouts or loss to follow-up.

**References:**

1. Manser T. Teamwork and patient safety in dynamic domains of healthcare: a review of the literature. Acta anaesthesiologica Scandinavica. 2009;53(2):143-51.

2. Körner M. Interprofessional teamwork in medical rehabilitation: a comparison of multidisciplinary and multidisciplinary team approach. Clinical rehabilitation. 2010;24(8):745-55.

3. Abdulrahman GO, Jr. The effect of multidisciplinary team care on cancer management. The Pan African medical journal. 2011;9:20.

4. Bach JA, Leskovan JJ, Scharschmidt T, Boulger C, Papadimos TJ, Russell S, et al. The right team at the right time - Multidisciplinary approach to multi-trauma patient with orthopedic injuries. International journal of critical illness and injury science. 2017;7(1):32-7.

5. Riemen AH, Hutchison JD. The multidisciplinary management of hip fractures in older patients. Orthopaedics and trauma. 2016;30(2):117-22.

6. Feng JE, Novikov D, Anoushiravani AA, Schwarzkopf R. Total knee arthroplasty: improving outcomes with a multidisciplinary approach. Journal of multidisciplinary healthcare. 2018;11:63-73.

7. Karjalainen K, Malmivaara A, van Tulder M, Roine R, Jauhiainen M, Hurri H, et al. Multidisciplinary biopsychosocial rehabilitation for neck and shoulder pain among working age adults. The Cochrane database of systematic reviews. 2003(2):Cd002194.

8. Letzel J, Angst F, Weigl MB. Multidisciplinary biopsychosocial rehabilitation in chronic neck pain: a naturalistic prospective cohort study with intraindividual control of effects and 12-month follow-up. European journal of physical and rehabilitation medicine. 2019;55(5):665-75.

9. Yanamadala V, Kim Y, Buchlak QD, Wright AK, Babington J, Friedman A, et al. Multidisciplinary Evaluation Leads to the Decreased Utilization of Lumbar Spine Fusion: An Observational Cohort Pilot Study. Spine. 2017;42(17):E1016-e23.

10. Namiranian K, Norris EJ, Jolissaint JG, Patel JB, Lombardi CM. Impact of Multidisciplinary Spine Conferences on Surgical Planning and Perioperative Care in Elective Lumbar Spine Surgeries. Asian spine journal. 2018;12(5):854-61.

11. Benton JA, Mowrey WB, De La Garza Ramos R, Weiss BT, Gelfand Y, Castro-Rivas E, et al. A Multidisciplinary Spine Surgical Indications Conference Leads to Alterations in Surgical Plans in a Significant Number of Cases: A Case Series. Spine. 2021;46(1):E48-e55.

12. Shang X, Huang Y, Li B, Yang Q, Zhao Y, Wang W, et al. Residents' Awareness of Family Doctor Contract Services, Status of Contract with a Family Doctor, and Contract Service Needs in Zhejiang Province, China: A Cross-Sectional Study. International journal of environmental research and public health. 2019;16(18).

13. Comino EJ, Davies GP, Krastev Y, Haas M, Christl B, Furler J, et al. A systematic review of interventions to enhance access to best practice primary health care for chronic disease management, prevention and episodic care. BMC health services research. 2012;12:415.

14. Shi L. The impact of primary care: a focused review. Scientifica. 2012;2012:432892.

15. Pertusa-Martínez S. [General Practitioners at the court of Queen Elizabeth II of England. Experience of a Spanish family doctor in the United Kingdom]. Atencion primaria. 2006;37(3):178-9.

16. Reyes-Llerena GA, Guibert-Toledano M, Penedo-Coello A, Pérez-Rodríguez A, Baez-Dueñas RM, Charnicharo-Vidal R, et al. Community-based study to estimate prevalence and burden of illness of rheumatic diseases in Cuba: a COPCORD study. Journal of clinical rheumatology : practical reports on rheumatic & musculoskeletal diseases. 2009;15(2):51-5.

17. Hung LM, Rane S, Tsai J, Shi L. Advancing primary care to promote equitable health: implications for China. International journal for equity in health. 2012;11:2.

18. Fu P, Wang Y, Liu S, Li J, Gao Q, Zhou C, et al. Analysing the preferences for family doctor contract services in rural China: a study using a discrete choice experiment. BMC family practice. 2020;21(1):148.

19. Liu S, Liu Y, Zhang T, Wang L, Huang J, Liang H, et al. The developing family doctor system: evidence from the progress of the family doctor signing service from a longitudinal survey (2013-2016) in Pudong New Area, Shanghai. BMC family practice. 2021;22(1):11.

20. Li J, Li J, Fu P, Chen Y, Tang X, Li Z, et al. Willingness of patients with chronic disease in rural China to contract with family doctors: implication for targeting characteristics. BMC family practice. 2021;22(1):203.

21. Hu J, Wang Y, Li X. Continuity of Care in Chronic Diseases: A Concept Analysis by Literature Review. Journal of Korean Academy of Nursing. 2020;50(4):513-22.

22. Bahr SJ, Weiss ME. Clarifying model for continuity of care: A concept analysis. International journal of nursing practice. 2019;25(2):e12704.

23. Ding C, Hong Y, Wang BY, Ning N, Chen JL, Yin ZW, et al. Expert consensus on the implementation of enhanced recovery after surgery in anterior cervical spine surgery. Chinese Journal of Bone and Joint Surgery. 2019;12(7):486-97.

24. Sun TS, Shen JW, Liu ZJ, Li CD, Hong Y, Sun CT, et al. Expert consensus in enhanced recovery after spinal surgery in China:perioperative management. Chinese Journal of Bone and Joint Surgery. 2017;10(04):271-9.

25. Zhou FF, Han B, liu L, Yuan L, Tian J, Li L, et al. Expert consensus on the implementation of enhanced recovery after surgery in posterior cervical spine surgery. Chinese Journal of Bone and Joint Surgery. 2019;12(07):498-508.
